# The Effects of Formaldehyde on Cytochrome P450 Isoform Activity in Rats

**DOI:** 10.1155/2017/6525474

**Published:** 2017-05-07

**Authors:** Min Xu, Huaqiao Tang, Qian Rong, Yuanli Zhang, Yinglun Li, Ling Zhao, Gang Ye, Fei Shi, Cheng Lv

**Affiliations:** College of Veterinary Medicine, Sichuan Agricultural University, Chengdu 611130, China

## Abstract

Formaldehyde (FA) is an occupational and indoor pollutant. Long-term exposure to FA can irritate the respiratory mucosa, with potential carcinogenic effects on the airways. The effects of acute FA poisoning on the activities of CYP450 isoforms CYP1A2, CYP2C11, CYP2E1, and CYP3A2 were assessed by determining changes in the pharmacokinetic parameters of the probe drugs phenacetin, tolbutamide, chlorzoxazone, and testosterone, respectively. Rats were randomly divided into three groups: control, low FA dose (exposure to 110 ppm for 2 h for 3 days), and high FA dose (exposure to 220 ppm for 2 h for 3 days). A mixture of the four probe drugs was injected into rats and blood samples were taken at a series of time points. Plasma concentrations of the probe drugs were measured by HPLC. The pharmacokinetic parameters *t*_1/2_, AUC_(0−*t*)_, and *C*_max_ of tolbutamide, chlorzoxazone, and testosterone increased significantly in the high dose versus control group (*P* < 0.05), whereas the CL of chlorzoxazone and testosterone decreased significantly (*P* < 0.05). However, *t*_1/2_, AUC_(0−*t*)_, and *C*_max_ of phenacetin decreased significantly (*P* < 0.05), whereas the CL of phenacetin increased significantly (*P* < 0.05) compared to controls. Thus, acute FA poisoning suppressed the activities of CYP2C11, CYP2E1, and CYP3A2 and induced the activity of CYP1A2 in rats. And the change of CYP450 activity caused by acute FA poisoning may be associated with FA potential carcinogenic effects on the airways.

## 1. Introduction

Formaldehyde (FA) is an occupational and indoor pollutant, with potential carcinogenic effects on the airways. The International Agency for Research on Cancer classified formaldehyde (FA) as a human carcinogen (Group 1) in 2004 [1]. FA is an occupational and indoor pollutant, which is widely employed in industries and also emitted from furniture, building materials, and chipboard [2]. Industrial applications range from resins and adhesives for wood-based materials and fiber insulation, to textiles, biocides, paper adhesives, timber covers, plastic, leather, building materials, and even cosmetics [3–5]. Residual FA can be found in leather, flooring, and new cars. Although the release of FA from these materials has been significantly reduced by manufacturers in recent years, indoor concentrations in prefabricated houses have not decreased by the same amount [6–9]. In fact, FA has long been a topic of environmental policy discussions as an air-polluting substance which enters the body primarily through respiration.

FA is not only a potent trigger of inflammation in the lower respiratory tract but also has a negative effect on other organs [10]. It is generally accepted that FA acts mainly at the site of first contact; however, it also affects metabolism in many different organs and presents in every cell of the human body [11]. Research has shown that animals inhaling FA exhibit increases in reactive oxygen species (ROS), malondialdehyde (MDA), and DNA-protein cross-linking and decreased levels of glutathione (GSH) in the lungs and systemically [12, 13]. FA mediated elevation in ROS and associated genotoxicity are implicated in the development of leukemia [14]. Other studies have shown that FA exposure is associated with elevated ROS/reactive nitrogen species (RNS) levels in the lungs as well as alterations in antioxidant enzyme concentrations [10]. After the oxidative damage caused by lipid peroxidation and protein biotransformation presented by individuals exposed to FA, there was recruitment of inflammatory cells [15]. In addition to the lungs, FA induced oxidative stress in the uterus and spleen [16–18]. Repeated treatments with 50 *μ*M FA changed the expression of more than 100 liver genes, showing that repeated inhalation of FA has widespread effects on major organs [19]. FA is metabolized to methanol and formic acid in the liver and erythrocytes after ingestion [20]. Current research on the toxicity of FA has in-depth damage in the liver, and FA induced liver injury model of research in recent years is also very mature [21, 22]. Such that Bakar et al. [23] found that FA markedly increased cell apoptosis in the liver.

The cytochrome P450 (CYP) enzyme system involved in metabolizing drugs in humans is predominantly expressed in the liver and intestine. Among the various CYP isozymes, CYP1A2, CYP2C9, CYP2C19, CYP2D6, and CYP3A4 are considered the most important metabolism enzymes [24–26]. The expression and activity of CYPs can be induced or suppressed by exogenous and endogenous materials; reactive oxygen intermediates produced in vivo and in vitro have been implicated in the cascade of events, leading to a loss of CYP during inflammation [27, 28]. As part of the vaccine manufacturing process, some residual FA (<0.02%) can be left behind, resulting in varying antibody responses to it [29, 30]. Environmental and occupational exposure, principally via inhalation, is a continuous focus in FA risk assessments. Because the toxic effect of FA can be found in several organs, including the liver, we speculated that liver CYPs might be altered by expose to FA.

## 2. Materials and Methods

### 2.1. Chemicals and Reagents

FA (>99.0%) was purchased from QILU SYNVA Pharmaceutical (Shandong, China). Phenacetin (>98.0%), tolbutamide (>99.0%), chlorzoxazone (>99.0%), testosterone (>98.0%), and the internal standard tinidazole (IS) (>99.0%) were purchased from Dalian Mellon Biological Technology (Dalian, China). High-performance liquid chromatography (HPLC) grade acetonitrile and methanol were purchased from Thermo Fisher Scientific (USA). Analytical-grade ethanol was purchased from Chengdu Kelong Chemical Reagent Factory (Chengdu, China).

### 2.2. Animals and Treatments

Twenty-four Sprague Dawley (SD) rats (220 ± 20 g, male) were obtained from Chengdu Dossy biological technology (Chengdu, China). Prior to the experiments, all animals were acclimatized for 7 days in a ventilated room, which was maintained at 25 ± 2°C and 70 ± 10% relative humidity with a 12-hour light/dark cycle. Thereafter, the rats were equally divided into three groups: control group, low-dose acute FA poisoning group, and high dose acute FA poisoning group. The rats were placed in a triad infected ark with a FA detector, and various concentrations of FA gas were passed through to create a model of acute FA poisoning. The high dose acute FA poisoning group was exposed to 220 ppm FA for 2 hours for 3 days; the low-dose group was exposed to 110 ppm FA for 2 hours for 3 days. Control animals were maintained under similar conditions, but without FA exposure. Rats were allowed to eat and drink ad libitum except during the 2 h exposure period. All animal experiments were performed in compliance with the Animal and Ethics Review Committee of Sichuan Agricultural University.

After FA treatment, a cocktail solution of the different probe drugs (50 mg/mL phenacetin, 25 mg/mL tolbutamide, 50 mg/mL chlorzoxazone, and 50 mg/mL testosterone in 45% ethanol-saline solution) was injected intraperitoneally to all rats at a dose of 1.0 mL/kg. Blood samples were collected at time points of 10, 20, 30, 40, 60, 120, 180, 240, 300, and 360 min through the fundus venous and immediately centrifuged at 6000 ×g for 10 min to obtain plasma. Then, 50 *μ*L plasma from each sample was stored frozen at −80°C until further analysis.

### 2.3. Sample Preparation for HPLC Analysis

To each 50 *μ*L plasma sample, 150 *μ*L tinidazole (100 mg/mL in acetonitrile) was added as the IS (standard tinidazole), vortexed for 1 min to mix. The samples were centrifuged at 8000 ×g for 10 min to obtain the supernatant (10 *μ*L), which were injected into the HPLC system for analysis.

### 2.4. Chromatographic Apparatus and Conditions

A 1260 Series liquid chromatograph (Agilent Technologies, Waldbronn, Germany), equipped with a quaternary pump, degasser, autosampler, thermostat controlled column compartment, and Agilent 1260 infinity variable wavelength detector was utilized for the analysis. The instrument was controlled by ChemStation software (Agilent Technologies, Santa Clara, CA). Chromatographic separation was achieved on a Zorbax SB-C18 column (Agilent Technologies, Germany) (4.6 × 250 mm, 5 *μ*m) with the column temperature set at 30°C. The mobile phase consisted of acetonitrile (A) and 0.01 M ammonium acetate in water (B). The gradient elution was 30–50% A over 0–15 min, holding at 50% A from 15–18 min and 50–30% A over 18–25 min. The flow rate, injection volume, and UV-detection wavelength were 1.0 mL/min, 10 *μ*L, and 230 nm, respectively. Quantification was performed by the peak-area method [31].

### 2.5. Statistical Analysis

The concentration-time profile of each probe drug was analyzed by DAS 2.0 (Wenzhou Medical College, Zhejiang, China). *T*-test was applied for statistical analyses using SPSS 19.0. A value of *P* < 0.05 or *P* < 0.01 was considered to be statistically significant. All analyses were repeated at least three times.

## 3. Results

### 3.1. Method Validation

**Figure 1:**
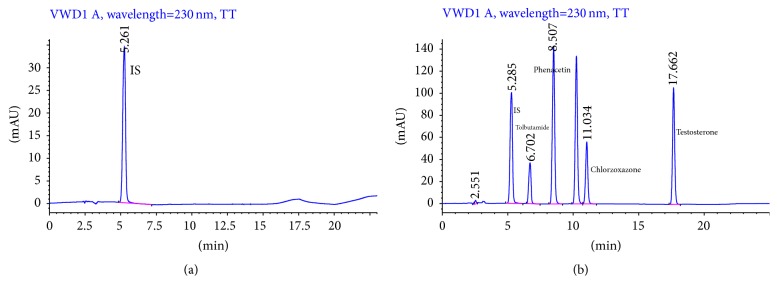
HPLC chromatogram of internal standard (IS) in the blank plasma and standard curves of the four probe drugs and IS added to the blank plasma.

To confirm the specificity and effectiveness of our HPLC analytical method, blank plasma was tested under the experimental conditions. There was no visible interference peak from the endogenous substances at the retention times for either IS or the probe drugs in the blank (Figure 1(a)) and sample plasma (Figure 1(b)). The retention times for IS, phenacetin, tolbutamide, chlorzoxazone, and testosterone were 5.28, 8.50, 6.70, 11.03, and 17.66 min, respectively (Figure 1(b)). To quantify the probe drug concentrations in the sample plasma, the ratio of drug peak area to IS was used. The calibration curves were linear in the determined concentration ranges (Table 1).

### 3.2. Effect of FA on CYP1A2 Activity

**Figure 2:**
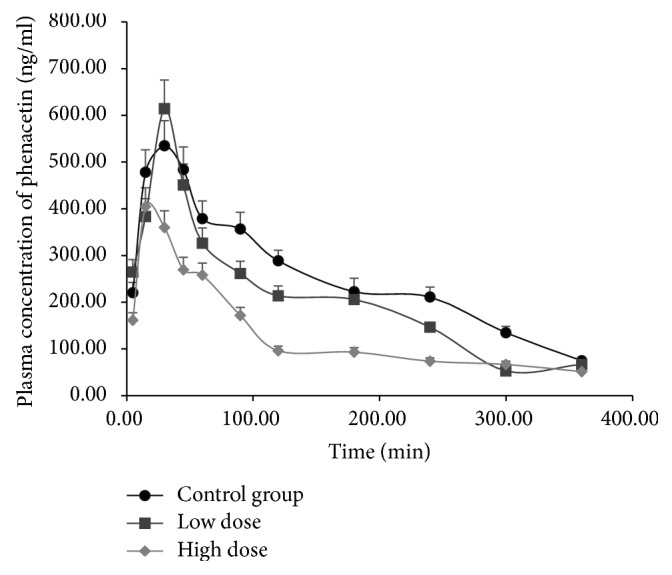
Mean plasma concentration-time curves of phenacetin in rats exposed to low or high dose of formaldehyde (FA).

Pharmacokinetic profiles of phenacetin in rats after FA treatment were used to describe the activity of CYP1A2. The effects of the different dosages of FA are presented in Table 2. Mean plasma concentration-time curves of phenacetin at different FA dosages are presented in Figure 2. After treatment with FA, *t*_1/2_, *V*_*d*_, and AUC_(0−*t*)_ of phenacetin decreased significantly compared to the control group; *C*_max_ of phenacetin decreased significantly only for high dose; however, CL increased significantly (*P* < 0.05; Table 2). In addition, the changes were dose-dependent. The results indicate that phenacetin metabolism in the treatment groups increases, and FA therefore has the potential to induce hepatic CYP1A2 activity in vivo.

### 3.3. Effect of FA on CYP2C11 Activity

**Figure 3:**
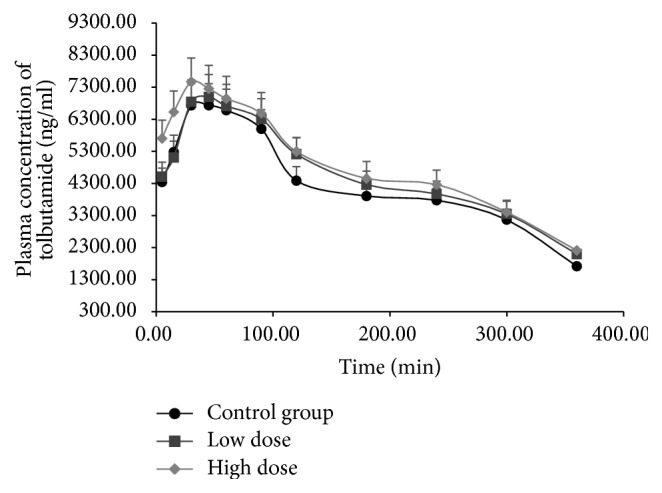
Mean plasma concentration-time curves of tolbutamide in rats exposed to low or high dose of formaldehyde (FA).

CYP2C11 activity was evaluated by the comparing pharmacokinetic behaviors of tolbutamide in the control and different FA treatment groups. Listed in Table 2 are the main pharmacokinetic parameters of tolbutamide. Mean plasma concentration-time curves of tolbutamide in different groups are presented in Figure 3. After treatment with FA, *t*_1/2_ of tolbutamide increased nonsignificantly (*P* > 0.05), whereas *V*_*d*_, AUC_(0−*t*)_, and *C*_max_ of tolbutamide increased significantly compared to control group (*P* < 0.05); the CL of tolbutamide did not change. These results demonstrate significant inhibition of CYP2C11 activity by FA in rats.

### 3.4. Effect of FA on CYP2E1 Activity

**Figure 4:**
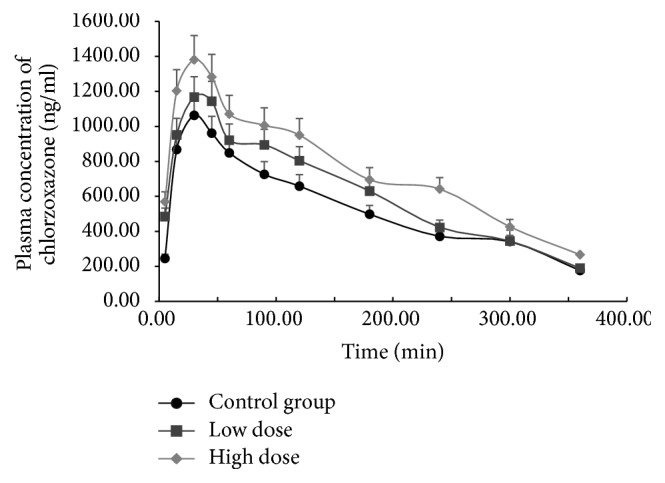
Mean plasma concentration-time curves of chlorzoxazone in rats exposed to low or high dose of formaldehyde (FA).

The effects of different FA treatments on the pharmacokinetic parameters of chlorzoxazone in rats are presented in Table 2. Mean plasma concentration-time curves of chlorzoxazone in the different groups are presented in Figure 4. Compared to the control group, the pharmacokinetic parameters *t*_1/2_, *V*_*d*_, AUC_(0−*t*)_, and *C*_max_ of chlorzoxazone increased significantly after treatment with a high dose of FA; only CL decreased significantly (*P* < 0.05) with a high dose of FA. Therefore, the pharmacokinetic behaviors of chlorzoxazone indicate that FA inhibits rat hepatic CYP2E1 activity in vivo.

### 3.5. Effect of FA on CYP3A2 Activity

**Figure 5:**
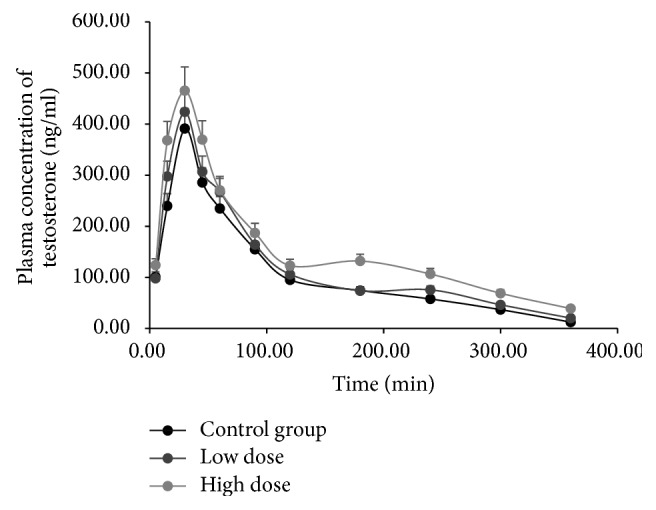
Mean plasma concentration-time curves of testosterone in rats exposed to low or high dose of formaldehyde (FA).

CYP3A2 activity was evaluated by comparing pharmacokinetic behaviors of testosterone between the control group and different FA treatment groups (Table 2). Mean plasma concentration-time curves of testosterone in the different groups are presented in Figure 5. After FA treatment, the pharmacokinetic parameters *t*_1/2_, AUC_(0−*t*)_, and *C*_max_ of testosterone in rats increased significantly in the high dose group compared to the controls, whereas CL of testosterone decreased significantly (*P* < 0.05); *V*_*d*_ decreased as well, albeit not significantly. The results indicate that a high dose of FA has the potential to inhibit rat hepatic CYP3A2 activity in vivo.

## 4. Discussion

FA is a ubiquitous occupational pollutant that is widely used in a variety of industries [32], as well as an indoor air pollutant [2]. Cardiovascular diseases, chronic respiratory disease, cancer, and hypertension are the main chronic, noncommunicable diseases in China [33]. These patients usually require long-term treatment, and they frequently come into contact with the widely distributed FA. If casual inhalation of FA can disrupt CYPs, FA may induce systematic oxidative stress and inflammation.

Sulfur dioxide (SO_2_) can cause toxicological damage to multiple organs in animals. The systemic oxidative damage produced by SO_2_ inhalation may influence or promote the progression or occurrence of disease states in various organs, and not only the respiratory system [34–37]. Furthermore, SO_2_ exposure can inhibit the activities and mRNA levels of CYP2B1/2, CYP2E1, and CYP1A1/2 in lungs and livers of rats [38, 39]. Studies have shown that hydrogen sulfide (H_2_S) toxicity is dependent on ROS production [40], and H_2_S exposure may inhibit the activities of CYP2B6, CYP2D6, CYP1A2, and CYP2C9 in rats [41]. At present, FA is widely used in various fields, and its toxic effects are of great concern. Like H_2_S, SO_2_ and other toxic gases, FA also affects CYPs. The results of this experiment show that FA exposure may inhibit the activities of CYP2E1, CYP2C11, and CYP3A2 and induce the activity of CYP1A2 in rats.

Compared to the control group, in the acute FA poisoning groups, *t*_1/2_, *V*_*d*_, AUC_(0−*t*)_, and *C*_max_ of phenacetin decreased significantly, but CL increased significantly (*P* < 0.05). *V*_*d*_, AUC_(0−*t*)_, and *C*_max_ of tolbutamide increased significantly compared to the control group (*P* < 0.05). *t*_1/2_, *V*_*d*_, AUC_(0−*t*)_, and *C*_max_ of chlorzoxazone increased (*P* < 0.01) after treatment with FA, whereas CL decreased significantly (*P* < 0.05). *t*_1/2_, AUC_(0−*t*)_, and *C*_max_ of testosterone increased significantly in high dose FA groups compared to controls (*P* < 0.05); CL of testosterone decreased significantly in the high dose group (*P* < 0.05), and *V*_*d*_ decreased as well, albeit not significantly (*P* > 0.05).

These results are important in terms of the effect of inhaling high amounts of FA on drug metabolism. After acute FA poisoning, drugs that are metabolized by CYP1A2, CYP2C11, CYP2E1, and CYP3A2 are likely to show interactions. Therefore, we should pay close attention to changes in their plasma concentrations. FA is a reducing agent, it is speculated that oxidative damage may be an important molecular mechanism of the toxic effect of FA, or maybe like H_2_S and SO_2_, it is caused a specific subtype gene expression changes and then changed the activity of particular subtype. The correlation research indicated [42, 43] that when exogenous substances cause CYP450 activity to change, it will cause activation of xenobiotic metabolism by cytochrome P450, production of oxidative stress, Nrf-2-mediated oxidative stress response, and oxidative DNA damage, subsequently, these may promote the development of cancer. Therefore, we hypothesized that the changes of CYP450 activity caused by acute FA poisoning may be associated with FA potential carcinogenic effects on the airways. Of course, this requires further testing to prove.

## 5. Conclusions

The concentrations of four probe drugs in rats plasma were successfully measured. In the experiment for acute FA poisoning and control group, there was a statistically significant increase in AUC_(0−*t*)_, *t*_1/2_, and *C*_max_ for tolbutamide, chlorzoxazone, and testosterone in FA groups while AUC_(0−*t*)_, *t*_1/2_, and *C*_max_ of phenacetin decreased significantly compared to the control group. In this experiment, we found that the activities of CYP2C11, CYP2E1, and CYP3A2 are inhibited, whereas that of CYP1A2 is induced following acute FA poisoning in rats. The results may make us pay close attention to the clinical oral use of drugs for the people after acute FA poisoning, and we speculate that the change of CYP450 activity caused by acute FA poisoning may be associated with FA potential carcinogenic effects on the airways.

## Figures and Tables

**Table 1:** Linear calibration parameters and detected concentration ranges of the probe drugs.

Analyte	Regression equation	*R* ^2^	Concentration range (*µ*g/mL)
Phenacetin	*y* = 176.97*x* + 1.0617	0.9968	0.25–250
Tolbutamide	*y* = 166.99*x* + 1.2795	0.9992	0.25–250
Chlorzoxazone	*y* = 128.95*x* + 1.3377	0.9996	0.25–250
Testosterone	*y* = 212.17*x* + 50.196	0.9985	0.25–250

**Table 2:** Main pharmacokinetic parameters of probe drugs in rats treated with formaldehyde (FA) (*n* = 8 per group, mean ± SD).

Probe drug name	Parameter	Control group	Low dose FA	High dose FA
Phenacetin	*t*_1/2_ (min)	163.89 ± 12.32	110.24 ± 13.19^*∗∗*^	92.42 ± 8.34^*∗∗*^
	CL (L/h/kg)	0.44 ± 0.11	0.61 ± 0.13^*∗*^	0.85 ± 0.09^*∗∗*^
	*V*_*d*_ (L/kg)	105.07 ± 9.78	96.51 ± 8.90	81.5 ± 7.89^*∗*^
	AUC_(0−*t*)_ (mg/mL*∗*min)	87.86 ± 8.56	71.7 ± 7.65^*∗*^	42.98 ± 6.09^*∗∗*^
	*C*_max_ (mg/L)	543.90 ± 35.06	614.13 ± 50.69^*∗*^	359.81 ± 31.24^*∗∗*^

Tolbutamide	*t*_1/2_ (min)	176.53 ± 16.76	181.24 ± 18.67	197.51 ± 16.45
	CL (L/h/kg)	0.01 ± 0.003	0.01 ± 0.002	0.01 ± 0.003
	*V*_*d*_ (L/kg)	9.09 ± 1.62	10.10 ± 1.31	13.98 ± 1.33^*∗∗*^
	AUC_(0 − *t*)_ (mg/mL*∗*min)	1481.73 ± 177.98	1592.93 ± 182.08	1660.67 ± 117.98^*∗*^
	*C*_max_ (mg/L)	6740.54 ± 289.07	7001.56 ± 302.01	7469.93 ± 346.78^*∗*^

Chlorzoxazone	*t*_1/2_ (min)	124.93 ± 16.43	133.92 ± 11.53	151.03 ± 11.90^*∗∗*^
	CL (L/h/kg)	0.23 ± 0.04	0.20 ± 0.05	0.16 ± 0.03^*∗*^
	*V*_*d*_ (L/kg)	41.16 ± 2.34	50.58 ± 3.55^*∗∗*^	57.32 ± 3.13^*∗∗*^
	AUC_(0 − *t*)_ (mg/mL*∗*min)	186.9 ± 12.34	216.75 ± 18.78^*∗*^	261.21 ± 21.33^*∗∗*^
	*C*_max_ (mg/L)	1063.40 ± 122.30	1167.62 ± 119.87	1381.03 ± 130.52^*∗*^

Testosterone	*t*_1/2_ (min)	80.27 ± 8.45	89.73 ± 10.09	92.91 ± 9.98^*∗*^
	CL (L/h/kg)	1.16 ± 0.12	1.11 ± 0.21	0.87 ± 0.18^*∗*^
	*V*_*d*_ (L/kg)	218.66 ± 18.76	213.40 ± 13.32	209.68 ± 12.78
	AUC_(0 − *t*)_ (mg/mL*∗*min)	37.85 ± 4.32	42.20 ± 3.22	52.91 ± 2.76^*∗∗*^
	*C*_max_ (mg/L)	391.24 ± 21.17	423.92 ± 26.74	465.39 ± 30.49^*∗*^

^*∗*^Significantly different from control, *P* < 0.05.

^*∗∗*^Significantly different from control, *P* < 0.01.
